# Disregarding multimappers leads to biases in the functional assessment of NGS data

**DOI:** 10.1186/s12864-024-10344-9

**Published:** 2024-05-08

**Authors:** Michelle Almeida da Paz, Sarah Warger, Leila Taher

**Affiliations:** Institute of Biomedical Informatics, Graz University of Technology, Graz, Austria

**Keywords:** Next-generation sequencing (NGS), ChIP-seq, RNA-seq, Multimappers, Functional analysis

## Abstract

**Background:**

Standard ChIP-seq and RNA-seq processing pipelines typically disregard sequencing reads whose origin is ambiguous (“multimappers”). This usual practice has potentially important consequences for the functional interpretation of the data: genomic elements belonging to clusters composed of highly similar members are left unexplored.

**Results:**

In particular, disregarding multimappers leads to the underrepresentation in epigenetic studies of recently active transposable elements, such as AluYa5, L1HS and SVAs. Furthermore, this common strategy also has implications for transcriptomic analysis: members of repetitive gene families, such the ones including major histocompatibility complex (MHC) class I and II genes, are under-quantified.

**Conclusion:**

Revealing inherent biases that permeate routine tasks such as functional enrichment analysis, our results underscore the urgency of broadly adopting multimapper-aware bioinformatic pipelines –currently restricted to specific contexts or communities– to ensure the reliability of genomic and transcriptomic studies.

**Supplementary Information:**

The online version contains supplementary material available at 10.1186/s12864-024-10344-9.

## Background

Next-generation sequencing (NGS) technologies such as Chromatin Immunoprecipitation followed by sequencing (ChIP-seq) [1] and RNA-seq [2, 3] have emerged as the state of the art for obtaining insights into gene regulatory processes. ChIP-seq and RNA-seq sequencing reads are typically short, with customary protocols recommending 1×50 bp and 2×75 bp, respectively [4, 5]. Such read lengths are insufficient to completely span many of the repetitive elements that abound in complex eukaryotic genomes. Consequently, standard analysis pipelines struggle to unambiguously trace the locus from which the reads have arisen and fail to quantify closely related sequences of the genome.

The challenge of assigning reads which map equally well to multiple loci in the genome has been discussed for over a decade. Already in the early days of NGS, Chung et al. [6] acknowledged that in ChIP-seq data 32% of human STAT1 and 74% of mouse GATA1 binding sites (“peaks”) were unlikely to be detected when ambiguously mapping reads (“multimappers”) were discarded from the analysis. Similarly, while trying to determine the range of detection of RNA-seq, Mortazavi et al. [7] found that 13–24% of the 25 bp-long reads obtained after sequencing transcriptomic libraries from mouse brain, liver and skeletal muscle tissues were multimappers, and suggested that discarding multimappers would result in a severe underestimation of genes with closely related paralogs, such as the members of the ubiquitin B family (97% of the reads that map to members of this family are multimappers). Furthermore, scientists working on the function and evolution of repetitive elements, particularly on transposable elements (TEs), have often expressed their concerns about most studies disregarding more than half of the human genome [8–10], and proposed several strategies to alleviate the problem [9, 11].

While various computational strategies have been proposed to mitigate the challenges posed by multimappers, to the best of our knowledge, no established NGS data processing pipeline offers an entirely satisfactory solution. In general, most strategies make prior assumptions about the distribution of the reads, and calculate the probability of a multimapper mapping to each of its possible target loci using a specific statistical model. Most strategies assume that multimappers and uniquely mapping reads (“unimappers”) are similarly distributed across the genome/transcriptome, and that loci/transcript segments with high unimapper coverage (e.g., [6, 7, 12–14]) or enriched for reads relative to, for example, what could be expected under a random distribution (e.g., [15]), are the most likely source of those multimappers. Some tools also incorporate information on the likelihood of sequencing errors and variations (e.g., [16]). Nevertheless, recent studies have shown that multimappers are concentrated into a few regions of the genome with especially poor unimapper coverage [17], and therefore their distribution does not match the distribution of unimappers. Consistent with this observation, to estimate the likelihood of a multimapper’s origin some tools rely solely on attributes such as the mapping quality of the reads (e.g., [17, 18]) or the sequence similarity between the potential loci of origin of the reads (e.g., [19]), as well as on the proportion of multimappers shared between the potential loci/transcript segments of origin (e.g., [19]). Moreover, substantial effort has been invested into developing strategies that make minimal or no prior assumptions about the data. These strategies acknowledge multimappers by distributing them equally among all loci (e.g., [20, 21]) or randomly selecting one of their mappings (e.g., filtering for the secondary alignment flag using samtools-view [22]), and have been used as an ultimate solution for resolving ties when the mapping quality scores among multimapper’s mappings are equally good (e.g., Bowtie2 with “-k” option [23]). A comprehensive review of available tools is out of scope of this work, and can be found in the literature (e.g., [9, 11]).

Typical repetitive elements in many genomes, including the human genome, include TEs, tandem repeats, and satellite and microsatellite DNA. But also members of certain gene families, such as the globin gene family, homeobox genes and the olfactory receptors, exhibit strong sequence similarity [24–26]. Consistently, it has been noted that the expression of highly repetitive members of the ubiquitin family [7] and HLA class II beta chain paralogues, specifically, *HLA-DRB5* [18], can be underestimated by the practice of discarding multimappers. Unfortunately, despite the evident issue, standard transcriptomic pipelines, including the ones introduced by the ENCODE Project Consortium [27], disregard multimappers by default [28]. Not surprisingly, 87% (27 out of 31) of the articles recently published in the high-impacted journals Nature, Nature Genetics, Science, and Cell that report on the findings of ChIP-seq or RNA-seq data analyses do not acknowledge multimappers, while the remaining ones only partially recognize them, for example, by considering at most 10 of the mapping loci or requiring a minimum mapping quality score (Additional file 1: Suppl. Table 1).

With the present study, we aim to draw attention to biases in the functional interpretation of NGS data that result from disregarding multimappers. We demonstrate the problem by comparing the strategy used by standard NGS pipelines (e.g., ENCODE Project Consortium [27]), which simply filter out multimappers, to simple “multimapper-aware” approaches [9]. Our contribution is not to provide a definitive solution for the problem, but rather, to demonstrate its potential functional-level implications. Specifically, we analysed 9 ChIP-seq and 16 RNA-seq datasets for a small but diverse group of human and mouse cell types and experimental conditions (i.e., targeted protein or histone modification for ChIP-seq data, different treatments and replicates for RNA-seq data). In conclusion, we urge for the implementation of strategies accounting for multimappers in NGS pipelines.

## Methods

### Literature search

A PubMed search was carried out to identify articles accounting for multimappers. The following terms were used for the query: (ChIP-seq[tiab] OR RNA-seq[tiab]) OR (ChIP-seq[MeSH Terms] OR RNA-seq[MeSH Terms]) AND (“Nature”[Journal] OR “Nat Genet.“[Journal] OR “Cell”[Journal] OR “Science”[Journal]). A filter for publication date was applied for the period from 2022/8/01 to 2023/8/31.

If not specified, we assumed that the tools had been run with default parameters (Additional file 1: Suppl. Table 1).

### Datasets

We selected four human and mouse datasets from the ENCODE Project data repository [27] for single-end ChIP-seq and pair-end RNA-seq with read (or read pair) length ranging from 50 to 101 bp (Additional file 1: Suppl. Table 2).

### Repeat annotation

Repeat annotation was obtained from the RepeatMasker track of the UCSC Genome Browser [29]. Immediately adjacent or overlapping annotations for TEs with the same “name” (“repName” in the RepeatMasker track) were merged. We further refer to all TEs with the same name as a TE “group”.

### Quality control and read mapping

Quality of raw ChIP-seq and RNA-seq samples was assessed using FASTQC v0.11.9 [30]. Reads were trimmed for adapters with Cutadapt 2.8 [31] and filtered with Trimmomatic v0.39 [32]. Bwa mem v0.7.17 [33] and BBMap v39.01 [34] were used to map reads against the human (GRCh38/hg38) and mouse (GRCm38/mm10) genome assemblies for ChIP-seq; STAR v2.7.10a [35] was used for RNA-seq. Gene annotations (GRCh38.p13 and GRCm38.p4) were obtained from GENCODE [36]. Duplicated reads were filtered out using PICARD v2.24.0 [37]. Reads mapping to non-chromosomal scaffolds and mitochondrial chromosome were excluded from the analysis of ChIP-seq samples. Only reads mapped in a proper pair were considered for RNA-seq data analysis; they were retrieved with SAMtools v1.10 [22]. The parameters used for each tool are listed in Additional file 1: Suppl. Table 3.

### TE group age

The oldest clade in which the TEs from a given group can be assumed to have been active was retrieved from Dfam (“Clades” column, [38]).

### TE group coverage

Bedmap v2.4.37 [39] was used to identify overlaps between the coordinates of read mappings and annotated TEs. Reads that mapped only once in the genome were considered “unimappers”; reads that mapped more than once were considered “multimappers”. Read coverage was computed for each TE group as:

$${C}_{K}=\sum_{k\in K}\sum_{r\in Q}\sum_{{r}_{i}\in {M}_{r}}\frac{{I}_{k}\left({r}_{i}\right)}{\left|{M}_{r}\right|}\left(\frac{{l}_{{k}_{{r}_{i}}}}{{L}_{r}}\right)$$

where *K* is the set of all copies of a TE group, $Q$ is the set of all reads in the library, ${M}_{r}$ is the set of all loci to which read *r* (of length ${L}_{r}$) mapped and $\left|{M}_{r}\right|$ is the size of that set, and ${l}_{{k}_{{r}_{i}}}$ is the number of nucleotides of the *i*th mapping of read $r_{i}$, overlapping with TE copy $k$ . For each mapping ${r}_{i}$ of *r*

$$I_k\left(r_i\right)=\left\{\begin{array}{ll}1,&if\;r_i\;\mathrm{overlaps}\;\mathrm{with}\;k\\0,&otherwise\end{array}\right.{.}$$

### Gene expression quantification

Multimappers were defined as read pairs (“fragments”) for which at least one read of the pair mapped more than once in the genome.

Standard gene expression quantification was performed with HTSeq-count (v2.0.2, [40]) using default parameters (“--nonunique none”), i.e., not accounting for multimappers. The expression value of a gene $g$ was defined as ${H}_{g}/{L}_{g}$, where ${H}_{g}$ is the count for gene $g$ assigned by HTSeq-count, and ${L}_{g}$ is the gene length as defined by its start and end coordinates in the R Ensembl BioMart database v2.54.0 [41].

To account for multimappers, we used a “multimapper-aware” strategy that counted fragments in genes based on the list of genes (“set S”) overlapping with the fragment mappings generated by HTSeq-count [42]. Specifically, gene counts were computed for each gene $g$ as:

$${C}_{g}=\sum_{f\in Q}\sum_{{f}_{i}\in {M}_{f}}\frac{{I}_{g}\left({f}_{i}\right)}{\left|{M}_{f}\right|}$$

where $Q$ is the set of all fragments in the library, ${M}_{f}$ is the set of all mappings in the transcriptome for fragment $f$ and $\left|{M}_{f}\right|$ is the size of that set, and for each mapping ${f}_{i}$ of *f*

$$I_g\left(f_i\right)=\left\{\begin{array}{ll}1,&\mathrm{if}\;f_i\;o\mathrm{verlaps}\;\mathrm{with}\;g\\0,&otherwise\end{array}\right.{.}$$

Note that if ${f}_{i}$ overlaps not only with $g$ but also with at least another gene, then ${I}_{g}\left({f}_{i}\right)=0$. This is the default behaviour of HTSeq-count (Additional file 3: Additional Material).

A gene $g$ was considered “expressed” if ${C}_{g}>0$. The multimapper-aware expression value of gene $g$ was defined as ${C}_{g}/{L}_{g}$.

Genes were considered under-quantified by HTSeq-count if $\frac{{C}_{g}/{L}_{g}}{{H}_{g}/{L}_{g}}>2$, where ${H}_{g}$ is the count for gene $g$ assigned by HTSeq-count.

Computations were repeated with simulated libraries constructed by trimming the 3’ end of the read pairs to 25, 50 or 75 bp with Cutadapt 2.8 [31].

### Functional analysis

The 50, 100 or 200 protein-coding genes with the highest expression values were subjected to functional analysis using the “compareCluster()” function of the R clusterProfiler package (v.4.6.0, [43]). Gene type was retrieved from R Ensembl BioMart database v2.54.0 [41].

### Gene set enrichment analysis (GSEA)

GSEA [44] was conducted on the fold-changes between the normalised counts for all protein-coding genes computed with HTSeq-count and those computed with our multimapper-aware strategy using the “GSEA()” function of the clusterProfiler with 10,000 permutations and the C7: Immunologic Signatures” collection from the Human Molecular Signatures Database (MSigDB) (v.2023.2, [45]). Gene counts were normalised with the trimmed mean of M-values (TMM) method, by calculating scaling factors using the “calcNormFactors()” and “cpm()” functions of the edgeR package (v.3.40.2, [46]) with default parameters.

### Differential expression analysis

Differential expression analysis was performed for the mouse RNA-seq dataset (Additional file 1: Suppl. Table 2) using the DESeq2 (v.1.38.3, [47]) R package. Specifically, the “DESeq()” function was used with default options to compare gene expression across all time points (1 h, 2 h, 4 h, 6 h) following lipopolysaccharide treatment, relative to the untreated control group (0 h). Gene counts from the multimapper-aware strategy were rounded to the nearest integer. Genes with a false discovery rate (FDR) lower than 0.05 and a log_2_ fold-change lower than -1 (down-regulated) or greater than 1 (up-regulated) were considered differentially expressed. The “lfcShrink()” function was used for fold-change shrinkage, using the “apeglm” method.

## Results

**Fig. 1:**
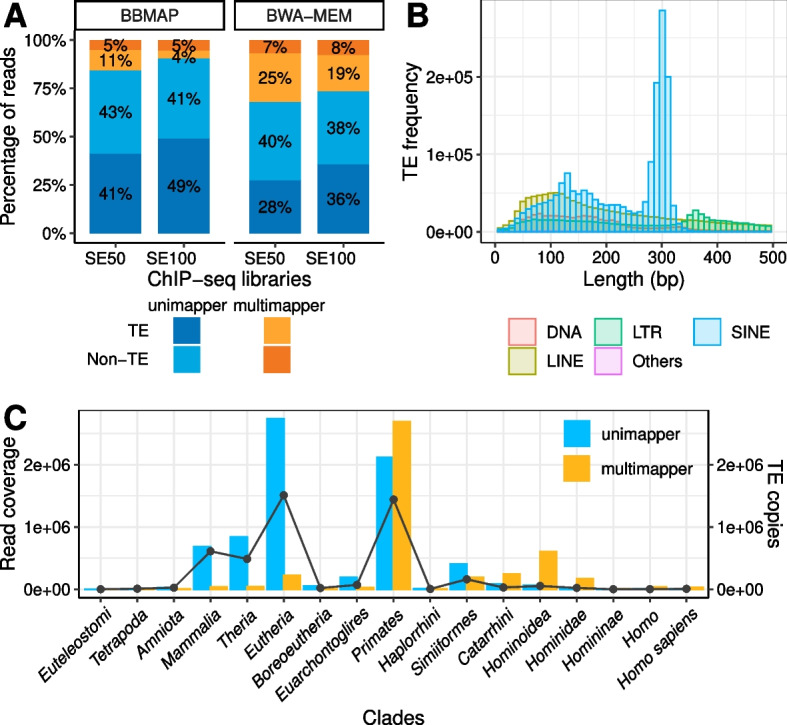
Discarding multimappers leads to epigenetic mischaracterization of young TEs. **A** Percentage of uni- and multimapper reads mapping to portions of the human genome annotated as TEs and not annotated as TEs (non-TE) for dataset 1 containing two ChIP-seq libraries generated by the ENCODE consortium using single-end 50 bp (“SE50”) and 100 bp (“SE100”) reads. Mapping was performed with two different mapping tools: with BBMap and Bwa mem. TEs are the major source of ChIP-seq multimappers in the human genome. **B** Length distribution of TE individual copies. Only TEs shorter than 500 bp are shown. Note that ~ 74% (856 out of 1,160) of the TEs in the human genome are longer than 500 bp, spanning up to 153,104 bp. The bin width is 10 bp. TEs were classified as DNA, LTR (long terminal repeat), SINE (short interspersed nuclear element), LINE (long interspersed nuclear element) and Others (e.g., rolling-circle (RC), unknown classification). Standard NGS reads are too short to fully cover most TE copies, explaining why TEs often give rise to multimappers. **C** Read coverage (bar plot on the left y-axis; see Methods) per clade for the SE100 ChIP-seq library (dataset 1) for uni- and multimappers. Reads were mapped using Bwa mem. TEs found in multiple clades (e.g., L1HS and L1P1) were assigned to the “younger” clade (*Homo* and *Hominoidea*, respectively). Only 30 out of 1,160 different TEs in the human genome (~ 3%) have not been annotated to any clade and were not represented. The number of TE copies for each clade (line plot on the right y-axis) shows that the majority of TEs are *Primates* and *Eutherian*-specific. Evolutionary young TEs are prone to be underrepresented when excluding multimappers from ChIP-seq analysis

Inspection of exemplary ChIP-seq ENCODE [27] libraries (Datasets 1 and 2; Additional file 1: Suppl. Table 2) revealed that multimappers constitute a substantial proportion (9–51%) of all reads mapped to the human genome, although the exact numbers vary greatly depending on the mapping tool –Bwa mem [33] (26–32%) reported twice or more the number of multimappers than BBMap [34] (9–16%) – and the immunoprecipitated protein (22–51%) (Additional file 2: Suppl. Fig. 1). Counterintuitively, when adhering to the current working standards and guidelines for ChIP-seq, we observed that the read length had only a relatively modest influence on the proportion of multimappers. Specifically, extending the read length from 50 to 100 bp resulted in a 17% reduction when utilising BWA mem and a 40% reduction with BBMap (Fig. 1A). As expected, a large fraction (43–80%) of multimappers mapped to regions annotated as TEs. Motivated by this fact and by the enormous expansion of repetitive TE sequences in mammalian genomes –they comprise ~ 46% of the human genome–, we used TEs to explore the impact of multimappers on an epigenetic analysis based on ChIP-seq data. TE individual copies in the human genome vary widely in length, from 10 (e.g., members of L2a) to 153,104 bp (nested LTR12B), but span a median of 231 bp, mostly reflecting the relatively recent expansion of elements from the SINE Alu family (median of 294 bp, Fig. 1B). Thus, although not all TEs give rise to multimappers, the large fraction of multimappers derived from TEs can be explained by the fact that TE copies are not fully covered by conventional NGS reads. In the datasets included in this study, at least 70% of the reads mapping to 8–16% (up to 181 out of 1,160) of TE groups are multimappers (Methods). Specifically, when considering every possible mapping, multimappers tended to be associated with evolutionary *young* TEs, such as AluYa5, L1HS and SVAs, while unimappers were associated with *old* TEs (*P*-value < 2.2 × 10^–16^, Chi-squared test; Fig. 1C; Additional file 2: Suppl. Figs. 2–4). And although with some deviations in TE group coverage (e.g., 3–55% for HERV-Fc1_LTR2), we made similar observations when considering only a random mapping for each multimapper (Additional file 1: Suppl. Table 4). This is natural, since relatively young TEs have not had enough time to accumulate variations in their sequences, but has far-reaching consequences: using standard ChIP-seq pipelines will specifically underrepresent recently active TEs, hampering their study. Nevertheless, TE activity is known to be associated with diverse human diseases [48], and hence, rectifying this issue promptly is imperative.

**Fig. 2:**
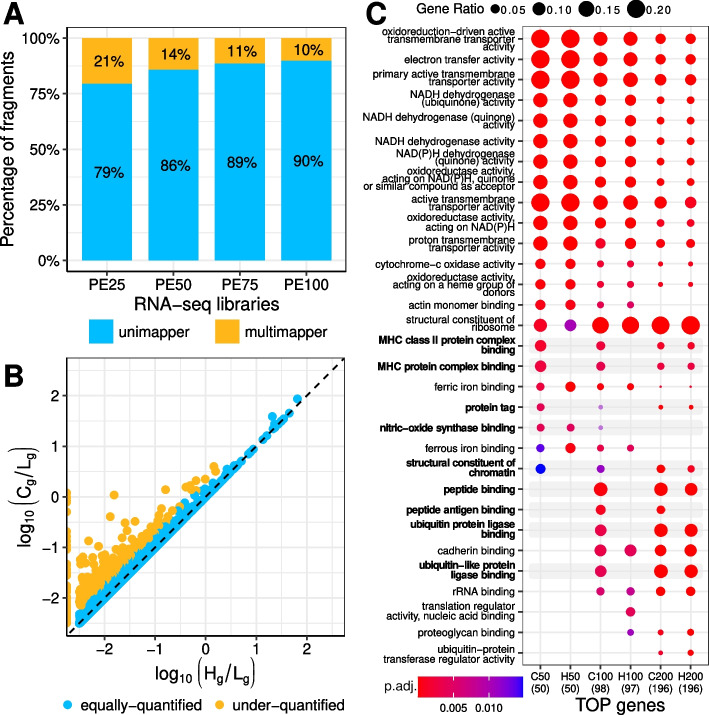
Discarding multimappers leads to functional mischaracterization of repetitive gene families. **A** Percentage of uni- and multimapper fragments mapping to human genome for a RNA-seq library of dataset 3 generated by the ENCODE consortium using pair-end 100 bp (“PE100”) and thereof simulated libraries with read pairs of length 25, 50 and 75 bp (“PE25”, “PE50” and “PE75”, respectively). Within the read lengths assessed, the difference in the proportion of multimappers was modest (10–21%). **B** Scatter plot showing gene expression values computed with HTSeq-count using default parameters (“–nonunique none”; x-axis) and by our “multimapper-aware” strategy (y-axis) for PE100. Each dot represents a protein-coding gene and is coloured differently depending on whether it is considered (approximately) equally-quantified or under-quantified by HTSeq-count (see Methods). The dashed line indicates identical gene expression values. About 6% (777 out of 13,437) expressed genes are under-quantified when discarding multimappers. **C** Gene ontology (GO) enrichment analysis of the 50, 100, and 200 protein-coding genes with the highest expression values in PE100 as computed by HTSeq-count (“H50”, “H100”, and “H200”, respectively) or our “multimapper-aware” strategy (“C50”, “C100”, and “C200”, respectively). GO enrichment analysis was performed for the “molecular function” category and using the “org.Hs.eg.db” annotation for the human genome. The q-value threshold was set to 0.01. Dot size represents the ratio between the number of genes in the given GO term (y-axis) and the number of genes annotated in each category (shown in brackets, below the label of each gene set on the x-axis). Dot colour indicates the *P*-value adjusted by Benjamini-Hochberg (BH, “p.adj.”). Neglecting multimappers leads to the underrepresentation of genes associated with specific GO terms (indicated in bold and with grey shading)

ChIP-seq is not the only NGS technology concerned by the current prevailing approach to handling multimappers. Standard RNA-seq bioinformatic pipelines use tools such as HTSeq-count [40] and STAR [35] for quantifying the reads mapping to annotated genes, and these tools also deliberately disregard multimappers. Although multimappers are not as abundant in RNA-seq data as they are in ChIP-seq data, they are not negligible. Using human and mouse RNA-seq dendritic cell libraries to illustrate the problem, we found that ~ 10% (Fig. 2A) and ~ 5% of the fragments mapped to the human and mouse genomes, respectively, were multimappers (Additional file 2: Suppl. Fig. 5). Similarly to what we observed for ChIP-seq, the read length had a relatively modest influence on the proportion of multimappers. More precisely, for paired-end RNA-seq, increasing the read length from 50 to 100 bp resulted in a 28% reduction. Moreover, analysis using HTSeq-count and STAR geneCounts with default parameters revealed quantification differences for about 6% (777 out of 13,437) of the human and 4% (468 out of 12,561) of the mouse genes expressed in these cells compared to a simple multimapper-aware strategy (Methods). Specifically, these genes were under-quantified by HTSeq-count and STAR geneCounts (Fig. 2B; Additional file 1: Suppl. Tables 5 and 6; Additional file 2: Suppl. Fig. 6), and most notably, they were not just random genes, but actually related to functions intrinsic to the biology of the samples under investigation, such as MHC class I and II immune responses and peptide antigen binding (Fig. 2C; Additional file 2: Suppl. Figure 7). GSEA analysis comparing the gene expression values from HTSeq-counts to those obtained with the multimapper-aware strategy further supported the association with perturbations of the immune system (Additional file 2: Suppl. Fig. 8). Perhaps more critically, these quantification biases can also impact the identification of differentially expressed genes. Indeed, we observed a substantial number of genes (9–81) that were exclusively differentially expressed when quantified with HTSeq-counts compared to the multimapper-aware strategy, or vice versa. Additionally, these genes were enriched for antigen presentation molecular functions (Additional file 2: Suppl. Figures 9 and 10). Naturally, RNA-seq dendritic cell libraries are no exception. Thus, for a collection of RNA-seq libraries of human lung carcinoma treated with three different drugs (i.e., dexamethasone, hydrocortisone or mapracorat), we found multimappers represented 6–9% of the fragments mapped to the genome, and 6–7% of the expressed genes were under-quantified when discarding multimappers (Additional file 2: Suppl. Fig. 11). In line with our findings in dendritic cells, accounting for multimappers resulted in differences in functional analysis, although in this case, the discrepancies were smaller (Additional file 1: Suppl. Table 7, Additional file 2: Suppl. Fig. 12 and 13). In essence, contingent upon the characteristics of the gene families expressed in the sample of interest, disregarding multimappers during the analysis of RNA-seq data may severely hinder the identification of critically relevant biological functions and processes.

## Discussion

For over a decade, scientists have grappled with the challenge of unambiguously assigning a substantial fraction of NGS short-reads to their original genomic loci. Most standard NGS pipelines filter out reads whose origin is ambiguous. Over time, numerous computational strategies have been proposed to acknowledge multimappers. All of them make prior assumptions, mostly about the distribution of reads in the NGS data (e.g., [6, 7, 12–14]). These assumptions have been naturally accepted as valid and only recently, it has been questioned whether they are valid [17]. To date, no gold-standard NGS pipeline exists that completely resolves the problem. It is up to the researchers to decide which assumptions are reasonable, and ultimately which biases are acceptable.

In this study, we investigated the implications of how multimappers are processed for the functional analysis of NGS data using two “multimapper-aware” approaches. One of the approaches accounts for multimappers by dividing the number of reads assigned to a locus/transcript segment by the total number of loci/transcript segments to which the reads map. The second approach randomly selects a mapping for each multimapper from the set of all multimapper’s mappings. The main advantage of these strategies is that they rely on a parsimonious set of assumptions, which makes them simple, intuitive and widely applicable. This alignment with Occam’s Razor reduces uncertainty and promotes generalizability. Meanwhile, the vast majority of strategies proposed in the literature allocate multimappers to a locus/transcript segment according to the distribution of unimappers or based on read mapping quality scores. While these strategies may seem more appealing and well-suited at first glance, they are flawed. First and foremost, the underlying assumptions are not necessarily true [17]. But even if multimappers could be assumed to be distributed as unimappers, and hence, the unimapper coverage served as a good indicator for inferring the loci of origin of multimappers, the low fraction of unimappers mapping to genomic elements like the members of the ubiquitin B family –only 3% of the reads mapping to these genomic elements are unimappers [7]– would make this inference unreliable. Similarly, the probability that a read is incorrectly mapped alone is insufficient for determining the locus of origin of a multimapper, as it can be easily influenced by other factors, such as sequencing errors and sequence variants. Naturally, despite their advantages, straightforward solutions to the problem such as the two “multimapper-aware” strategies we employed also have their limitations, as they may not comprehensively capture the entirety of the complexity of the problem. In particular, the approaches we adopted are suitable for calculating read coverage at the level of TE group/gene families, but do not allow to quantify how much a TE copy/transcript is expressed in comparison to other TE copies/transcripts of the same TE group/gene family [11].

Using exemplary random ChIP-seq and RNA-seq datasets, we showed that discarding multimappers can lead to biases in functional genomic/transcriptomic analyses. As might be expected, the magnitude of the biases varies with the dataset and the impact is more pronounced when shorter reads are used. Prevailing NGS platforms like Illumina produce massive quantities of highly accurate sequencing reads, but these reads are relatively short. Generally, we noted that the proportion of multimappers is determined by the read length. However, consistent with previous observations [49, 50] we found that the use of longer or paired-end Illumina reads does not result in substantial differences. Moreover, our findings suggest that the fraction of multimappers mapped to regions annotated as TEs and members of repetitive gene families also depends on the mapping tool (previously observed by [51]), targeted proteins or histone modifications, and treatment. Furthermore, also the identity of the TEs and genes most affected by the way multimappers are handled depends on the aforementioned factors, suggesting that the biases stemming from the practice of discarding multimappers may vary in severity, contingent upon the underlying biological context. Finally, it is important to note that the datasets illustrating the issue were chosen randomly. While an examination of a broader range of datasets may be warranted to unveil more nuanced trends, there is no compelling evidence to undermine the robustness of our general conclusion—that neglecting multimappers introduces biases in the functional analysis of NGS data.

Interpreting the functional significance of expressed (or differentially expressed) genes is often a primary goal in RNA-seq analysis. When genes exhibit sufficiently high sequence similarity, the practice of discarding multimappers is likely to affect the quantification of paralogous gene families, genes with internally repeated domains, and multiple isoforms of the same gene. Notable examples of such groups of genes include HLA class I (e.g., *HLA-B*, *HLA-E*) and class II (e.g., *HLA-DRA*, *HLA-DPA1*), polyubiquitin genes (e.g., *UBB*, *UBC*), chromatin (e.g., *MRNIP*) and cytoskeleton (e.g., *TUBB, TUBB2B*) components, and the recently discovered *BOLA2B* genes. It is worth mentioning that biases in gene quantification impact differentially expression analysis as well, potentially leading to both false positives and false negatives. These effects are likely to be exacerbated if poorly expressed genes are filtered out before testing for differential expression, a common practice.. Since in this study we have only focused on mRNA, we anticipate that many other types of RNA (e.g., miRNAs, rRNAs, tRNAs) present in multiple copies might be underestimated when discarding multimappers. Importantly, functional analysis may not always reveal underrepresented functions, as we found for dendritic cell libraries, in which functions related to adaptive immunology were well associated with multimappers, but can be minor, as for the analysed lung cancer libraries. Researchers had previously identified isolated instances of genes exhibiting varying quantification results depending on how multimappers were handled. Here, we demonstrate that these effects extend beyond individual genes and manifest at the functional level.

Despite its intuitive nature, the problem posed by multimappers and their impact on functional NGS analysis are routinely disregarded by standard bioinformatics pipelines. This oversight results in the neglect of clusters of repetitive genomic elements with highly similar members. We therefore believe that addressing the problem outlined in this study may entail applying one or more multimapper-aware strategies and contrasting their results with those of strategies that do not account for multimappers. Furthermore, emerging NGS technologies such as PacBio and Oxford Nanopore enable the acquisition of ultra-long reads, having already reached the impressive mark of more than 2 Mbp [52], and thus, hold the potential to substantially reduce the number of ambiguously mapping reads. However, they are currently limited by their higher cost and lower accuracy when compared to Illumina NGS. An alternative to achieve the desired outcome could be combining these two technologies. Ultimately, it becomes imperative that new computational guidelines acknowledging multimappers are established and disseminated by major projects.

## Conclusions

Our research shows that neglecting multimappers during NGS data processing can have a substantial impact on biological inferences drawn from genomic and transcriptomic data. To the best of our knowledge no other article has explored the functional-level implications of this practice embedded in the ENCODE guidelines. Notably, we showed that the issue extends beyond specific scientific communities, such as those dedicated to the study of TEs. Indeed, our findings emphasise that even a seemingly routine task such as performing a gene ontology (GO) enrichment analysis on any given RNA-seq dataset can be susceptible to biases. And consequences are far-reaching: candidates identified for further functional assays may be considerably suboptimal.

### Supplementary Information

**Additional file 1. **Supplementary Tables 1–7.

**Additional file 2. **Supplementary Figs. 1–10.

**Additional file 3. **Additional Material and Additional Figs. 1–20.

## Data Availability

ChIP-seq and RNA-seq datasets were acquired, processed, and analysed from datasets described in Additional file 1: Suppl. Table 2. Code generated and used during the current study are available from the corresponding author on reasonable request.
