# Supplementary material for: Disregarding multimappers leads to biases in the functional assessment of NGS data
Source: BMC Genomics. 2024 May 8;25:455. doi: 10.1186/s12864-024-10344-9 (PMC11078754; doi:10.1186/s12864-024-10344-9)
Supplement: Supplementary file 2 — Additional file 2. Supplementary Figs. 1–10. [file 12864_2024_10344_MOESM2_ESM.pdf]

Discarding multimappers leads to biases in the functional assessment of NGS data

Michelle Almeida da Paz

michelle.almeidadapaz@tugraz.at

Sarah Warger

sarah.warger@student.tugraz.at

Leila Taher (corresponding author)

leila.taher@tugraz.at

Institute of Biomedical Informatics, Graz University of Technology, Graz, Austria

Supplementary Figures

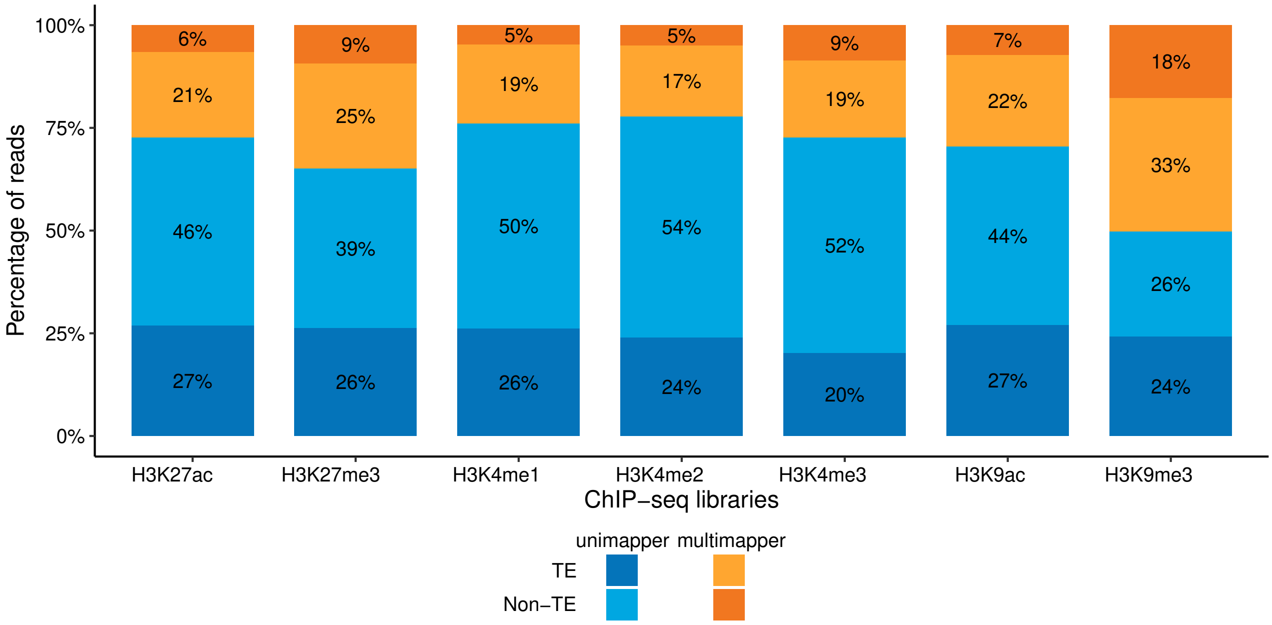

Supplementary Figure 1. Percentage of uni and multimapper reads for dataset 2. Libraries were generated by the ENCODE consortium using single-end 50 bp. Reads were mapped to the human genome using BWA-MEM. For complete description, see legend of Figure 1.

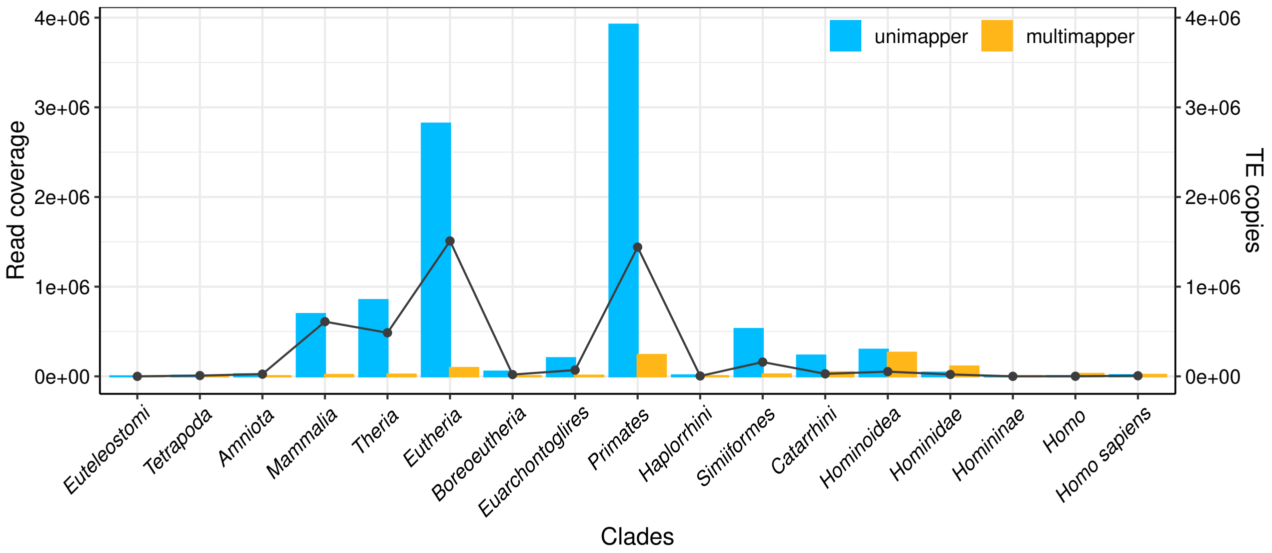

Supplementary Figure 2. Human SE100 ChIP-seq library mapped using BBMAP. Read coverage (left y-axis) and number of TE copies (right y-axis) per clade for uni- and multimappers. For complete description, see legend of Figure 1.

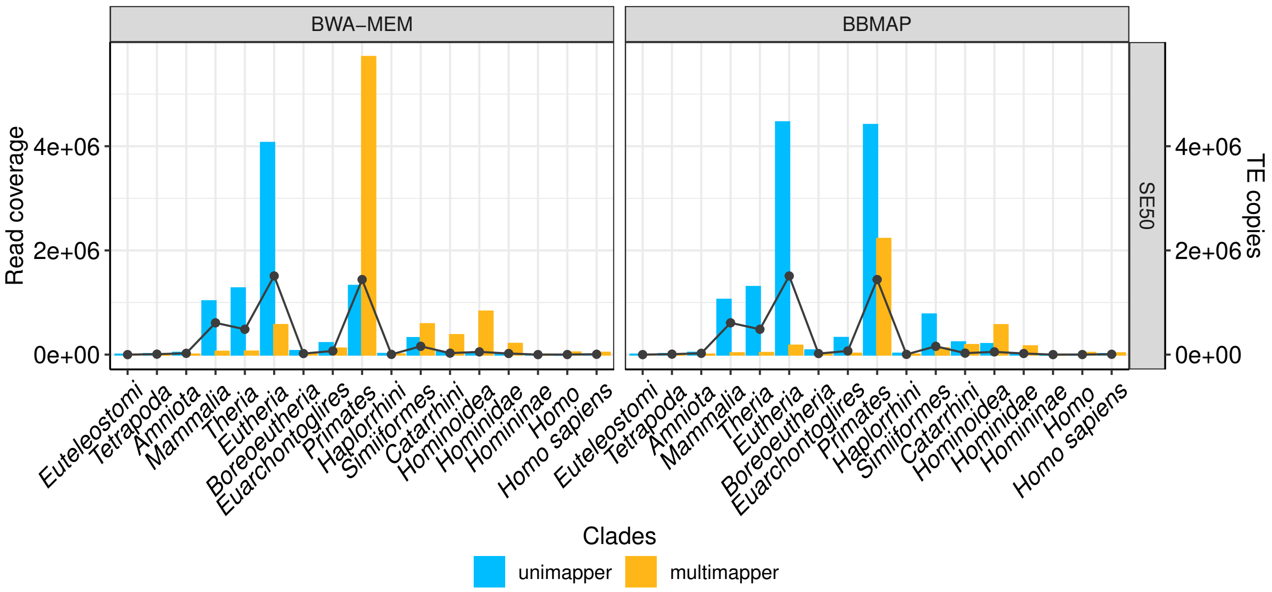

Supplementary Figure 3. Human SE50 ChIP-seq library mapped using BWA-MEM and BBMAP. Read coverage (left y-axis) and number of TE copies (right y-axis) per clade for uni- and multimappers. For complete description, see legend of Figure 1.

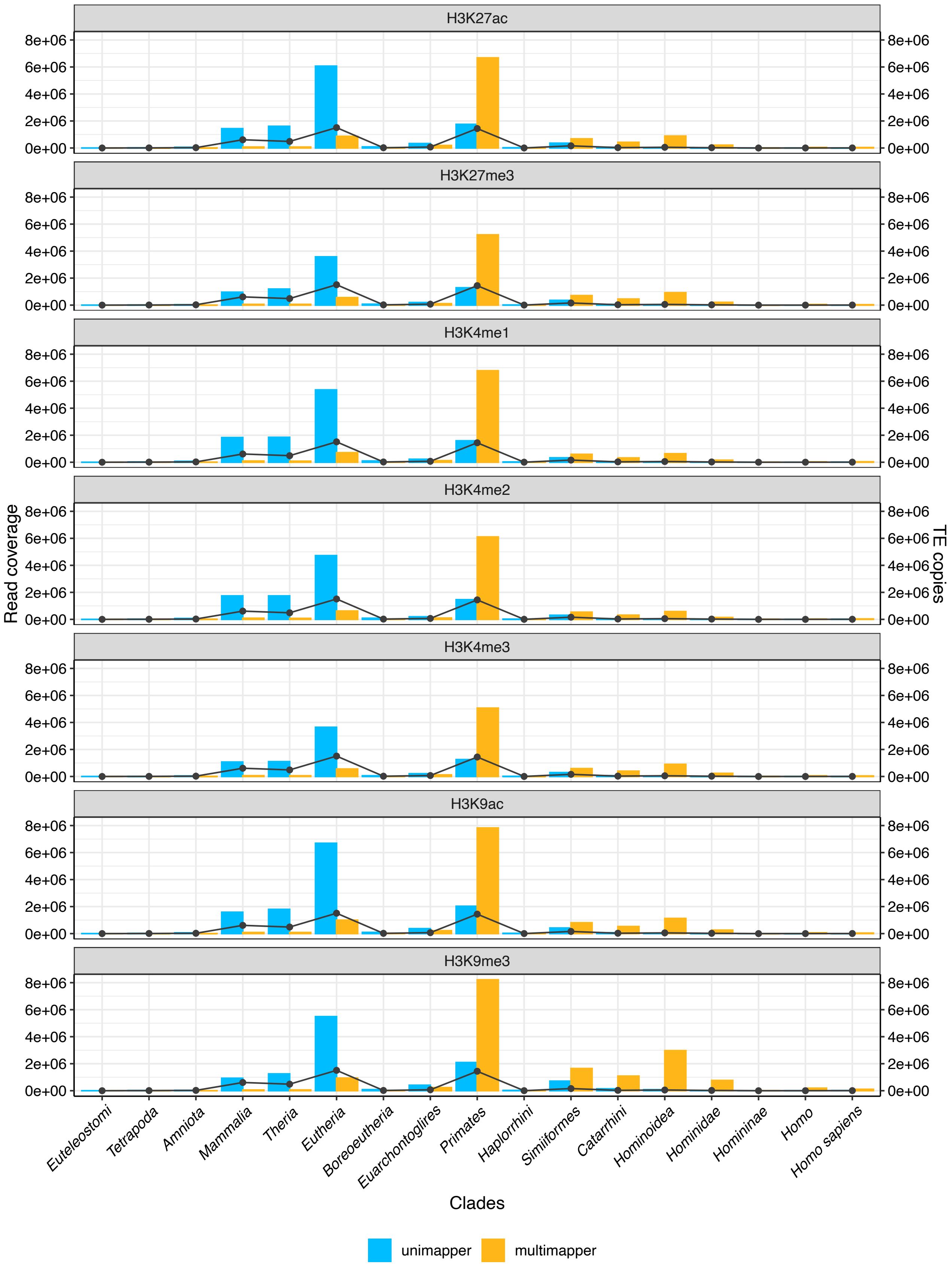

Supplementary Figure 4. ChIP-seq libraries from dataset 2 mapped using BWA-MEM. Read coverage (left y-axis) and number of TE copies (right y-axis) per clade for uni- and multimappers. For complete description, see legend of Figure 1.

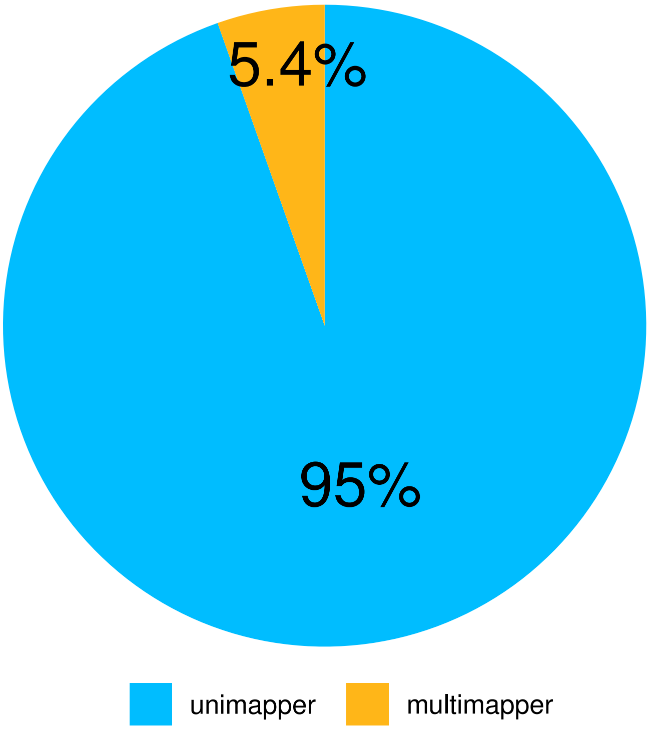

Supplementary Figure 5. Percentage of uni- and multimapper fragments for mouse PE75 RNA-seq library. Library was generated by the ENCODE consortium using pair-end 75 bp. Fragments were mapped to the mouse genome.

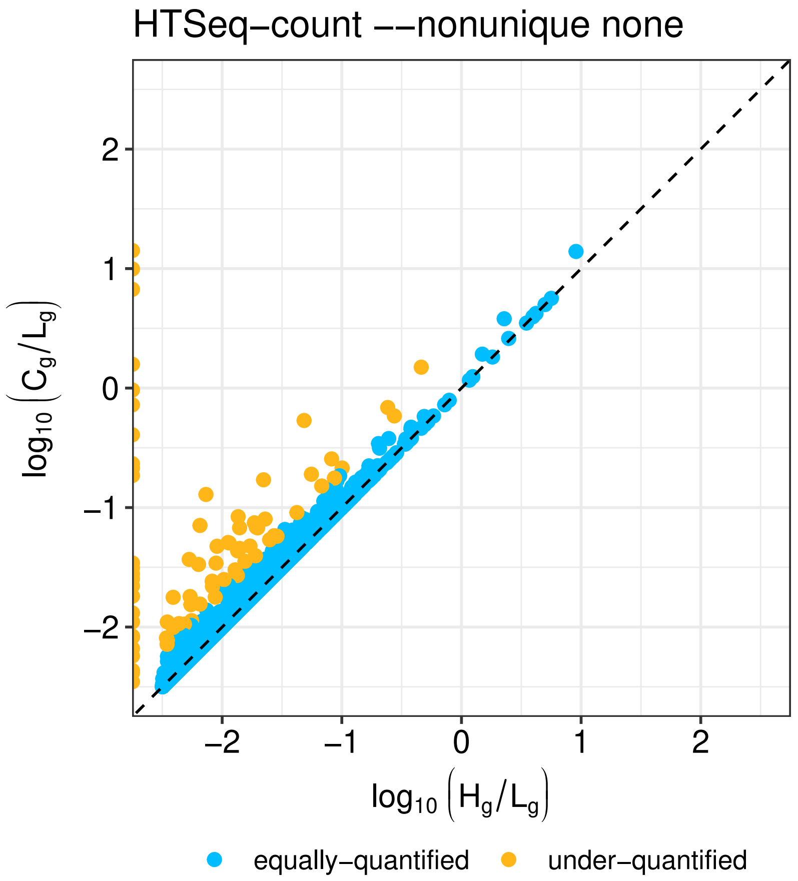

Supplementary Figure 6. Gene under-quantification by HTSeq-count (--nonunique none) for mouse PE75 RNA-seq library. Scatter plot showing under-quantified protein-coding genes by HTSeq-count using default parameters ("--nonunique none"; x-axis), when comparing to "multimapper-aware" expression values (y-axis). About 4% (468 out of 12,561) expressed genes are under-quantified when discarding multimappers. For complete description, see legend of Figure 2B.

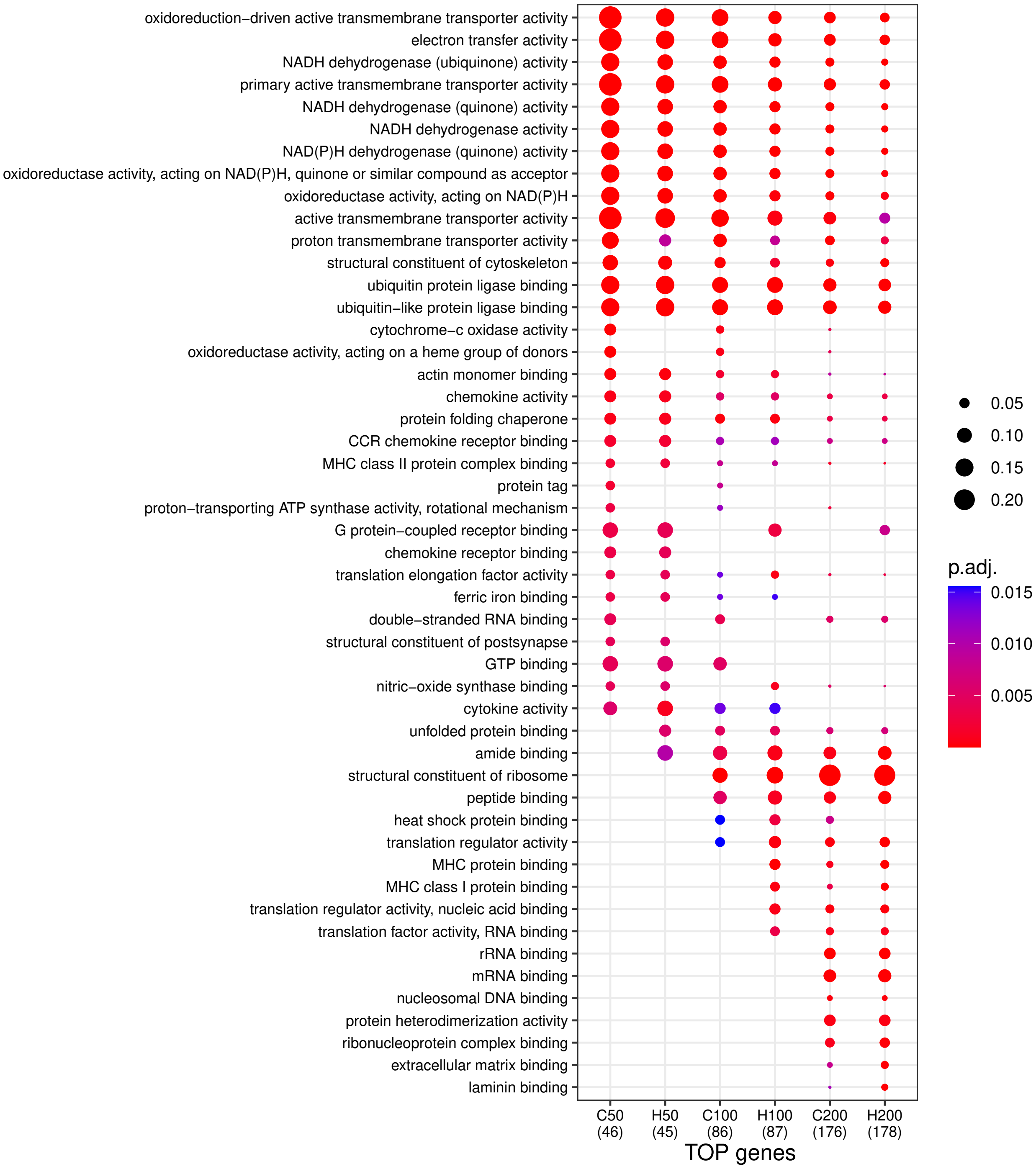

Supplementary Figure 7. Functional underrepresentation by HTSeq-count (--nonunique none) for mouse PE75 RNA-seq library. Dot plot showing gene ontology (GO) enrichment analysis of the 50, 100, and 200 protein-coding genes with the highest expression values as computed by HTSeq-count using default parameters ("--nonunique none") ("H50", "H100", and "H200", respectively) or our "multimapper-aware" strategy ("C50", "C100", and "C200", respectively). For complete description, see legend of Figure 2C.

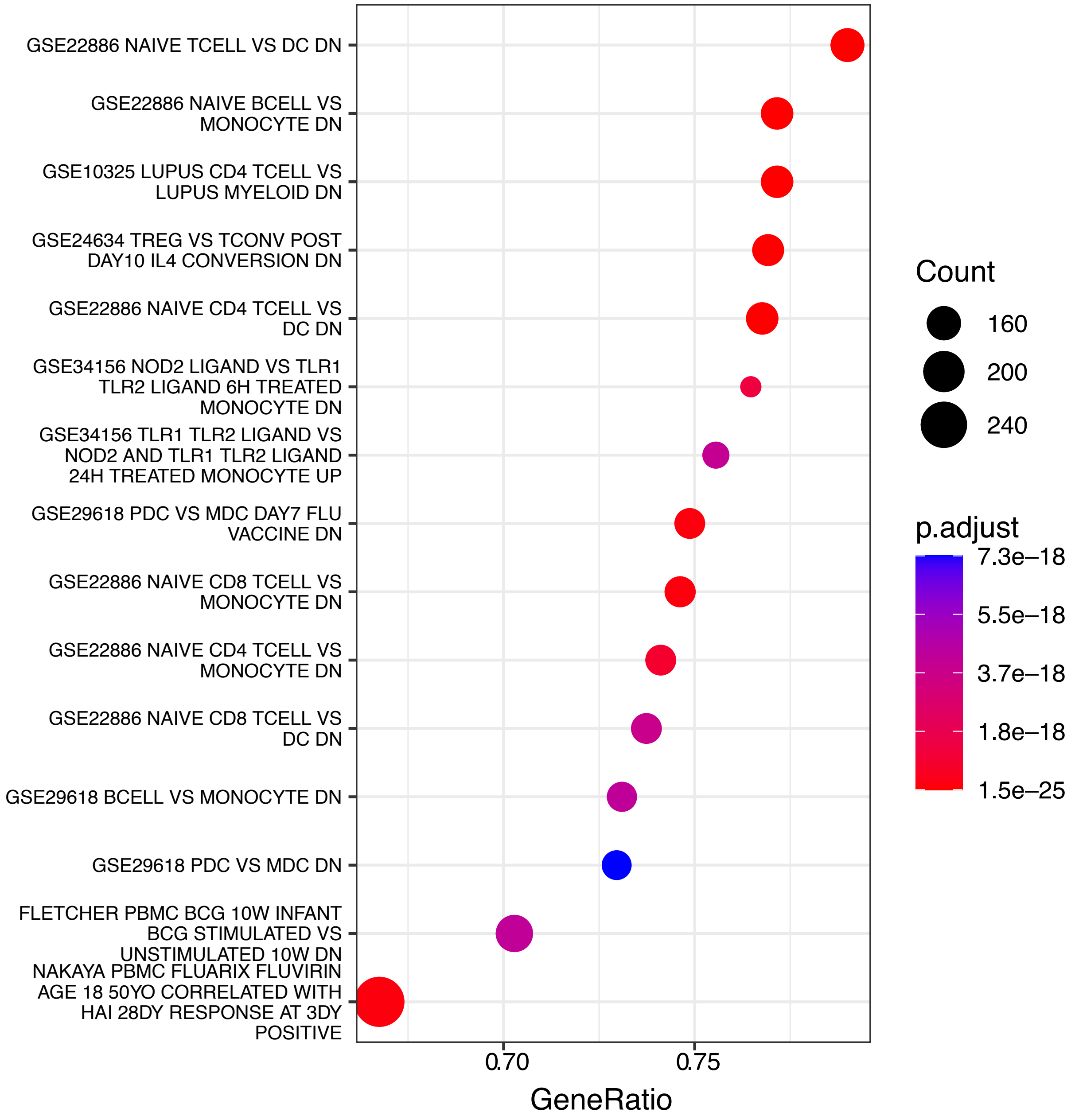

Supplementary Figure 8. Gene set enrichment analysis (GSEA) comparing gene expression levels obtained with HTSeq-count (--nonunique none) and a multimapper-aware strategy for human dendritic cells (PE100 RNA-seq library; Additional file 1: Suppl. Table 2). The size and colour intensity of a circle represents the numbers of genes and adjusted P-value for each gene set, respectively. We observed differences in 835 out of 5,319 genes sets related to the immune system (adjusted P-value < 0.05). Only 15 enriched gene sets are displayed.

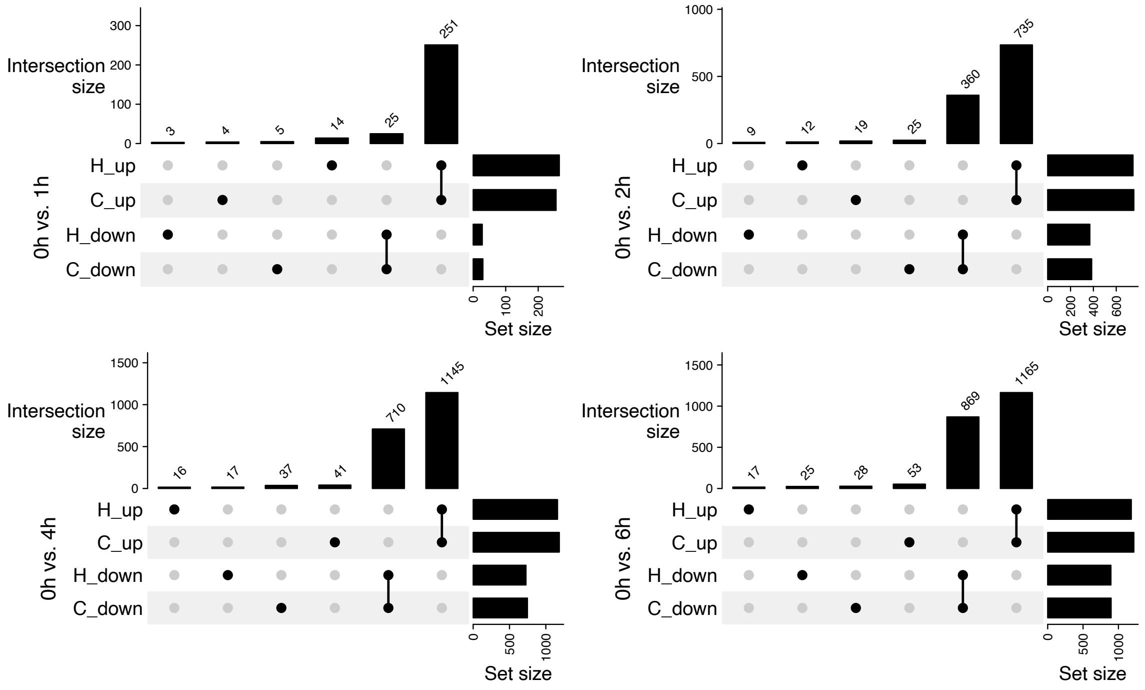

Supplementary Figure 9. Upset plots for the differential expression analysis across several time points (1h, 2h, 4h, 6h) following lipopolysaccharide treatment relative to control (0h) of mouse dendritic cells (PE75 RNA-seq library; Additional file 1: Suppl. Table 2). Comparison between the numbers of up- ($\log_2$ fold-change>1) and down- ($\log_2$ fold-change<-1) differentially expressed (FDR < 0.05) protein-coding genes for HTSeq-count ("H", --nonunique none) and our multimapper-aware strategy ("C").

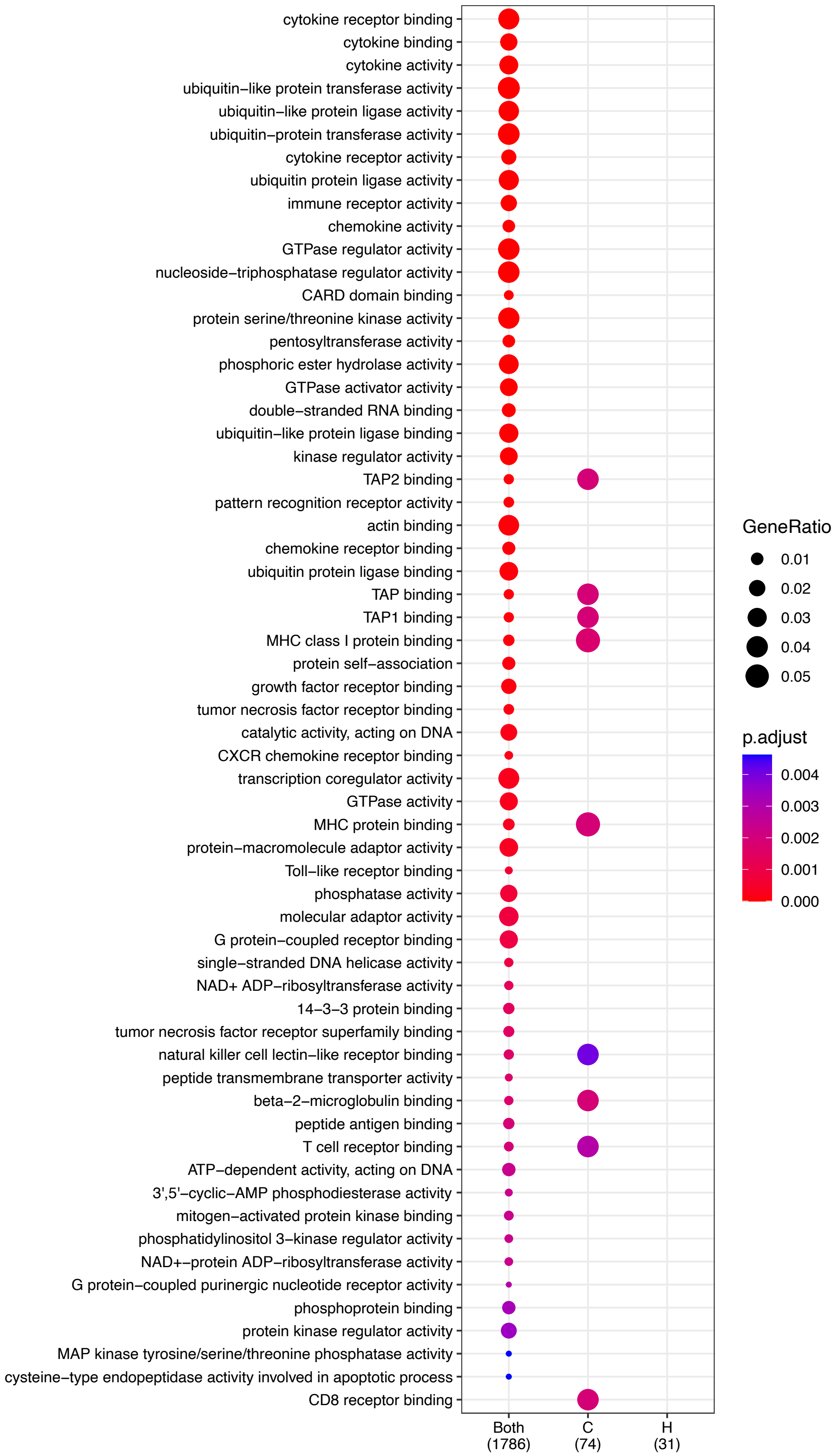

Supplementary Figure 10. Functional analysis of time points 4h versus 0h for mouse PE75 RNA-seq library. Gene ontology (GO) enrichment analysis of the 1786 differentially expressed protein-coding genes in HTSeq-count (--nonunique none) and our multimapper-aware strategy ("Both"); 74 differentially expressed genes exclusively for the multimapper-aware strategy ("C") and 31 differentially expressed genes exclusively for the HTSeq-count. For complete description, see legend of Figure 2C.

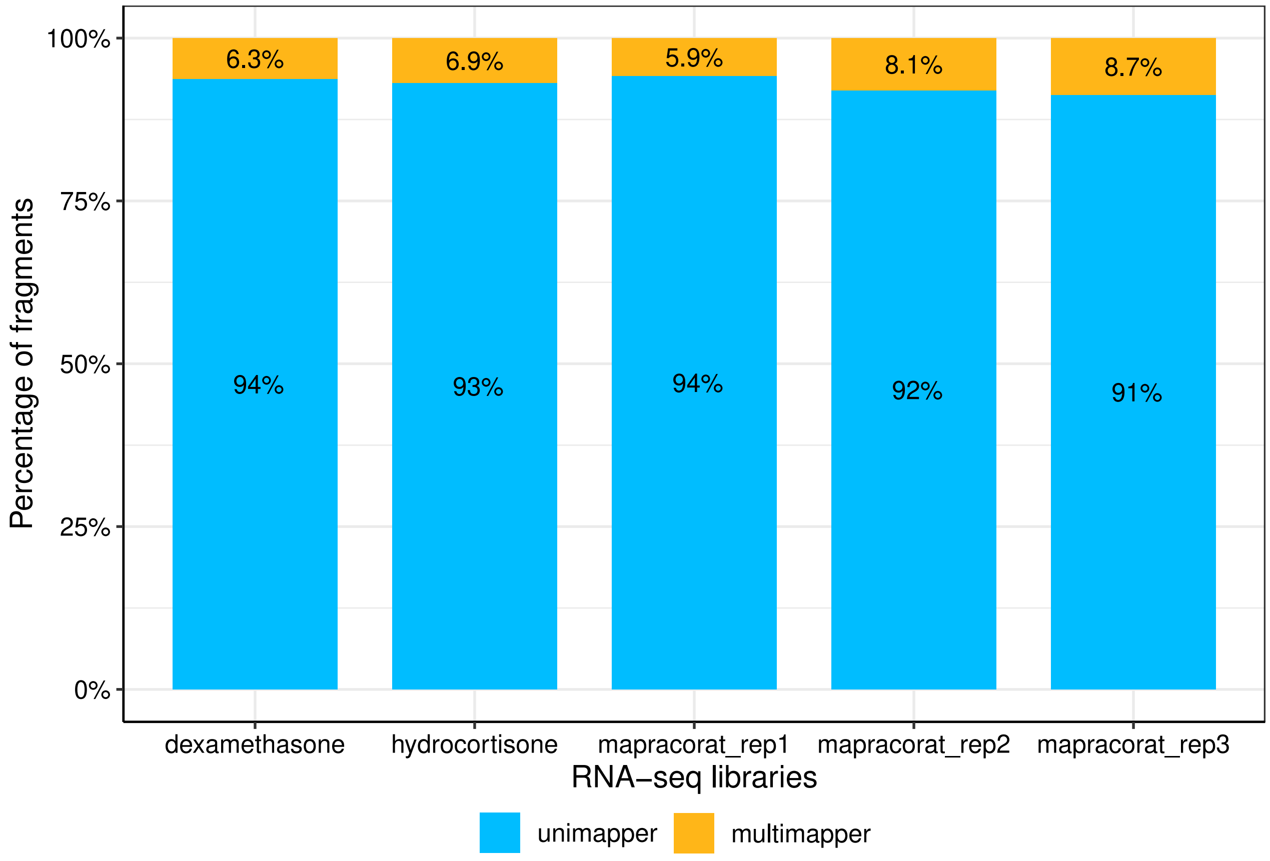

Supplementary Figure 11. Percentage of uni and multimapper fragments for dataset 4. Libraries were generated by the ENCODE consortium using pair-end 101 bp. For complete description, see legend of Figure 2.

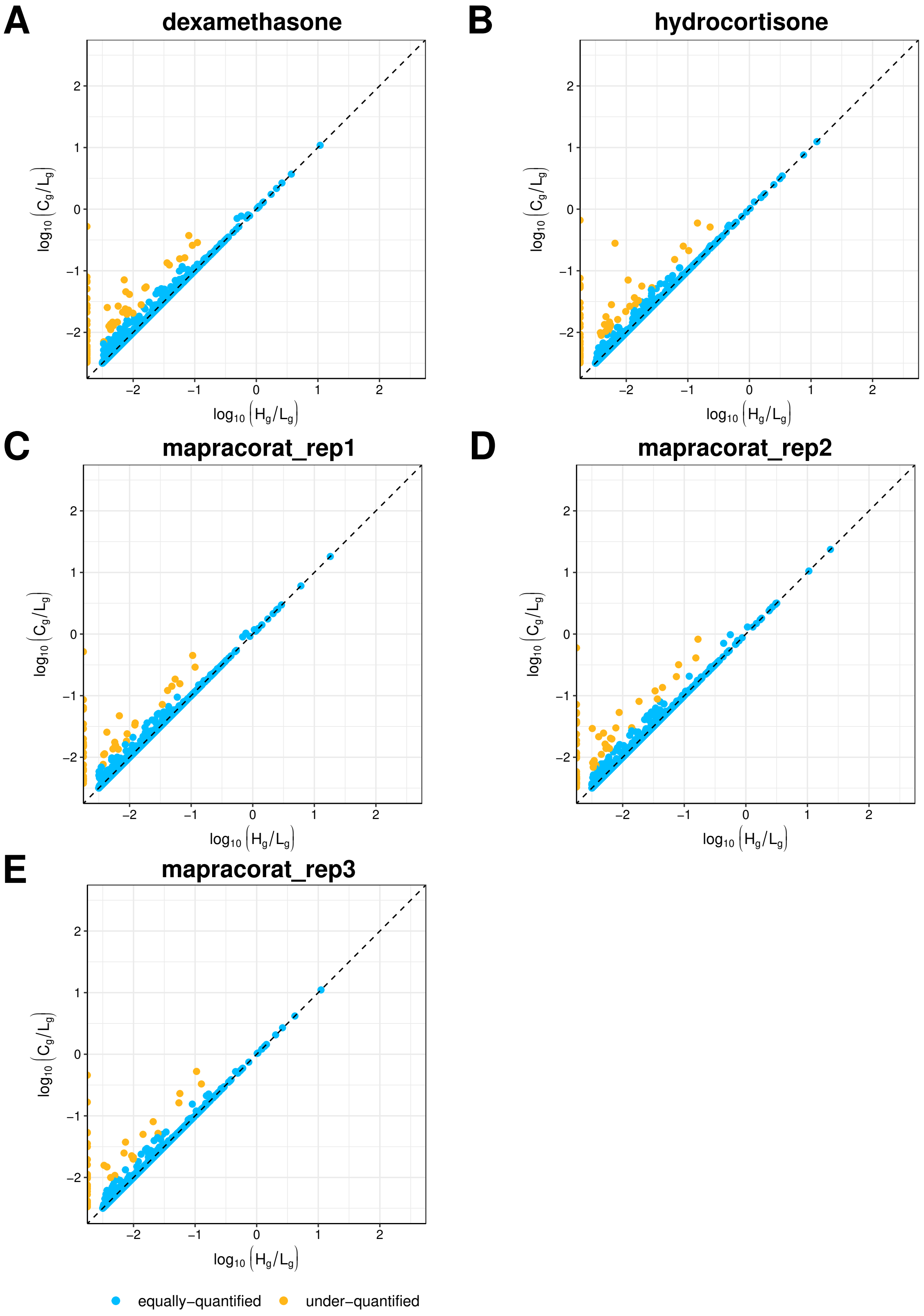

Supplementary Figure 12. Gene under-quantification by HTSeq-count (--nonunique none) for RNA-seq libraries of dataset 4. Scatter plot showing under-quantified protein-coding genes by HTSeq-count using default parameters ("--nonunique none"; x-axis), when comparing to "multimapper-aware" expression values (y-axis). Percentage of expressed genes that are under-quantified when discarding multimappers for **(A)** Dexamethasone: 6% (834 out of 13,295). **(B)** Hydrocortisone: 7% (865 out of 12,826). **(C)** Mapracorat – replicate 1: 6% (817 out of 12,942). **(D)** Mapracorat – replicate 2: 7% (883 out of 13,190). **(E)** Mapracorat – replicate 3: 7% (831 out of 12,101). For complete description, see legend of Figure 2B.

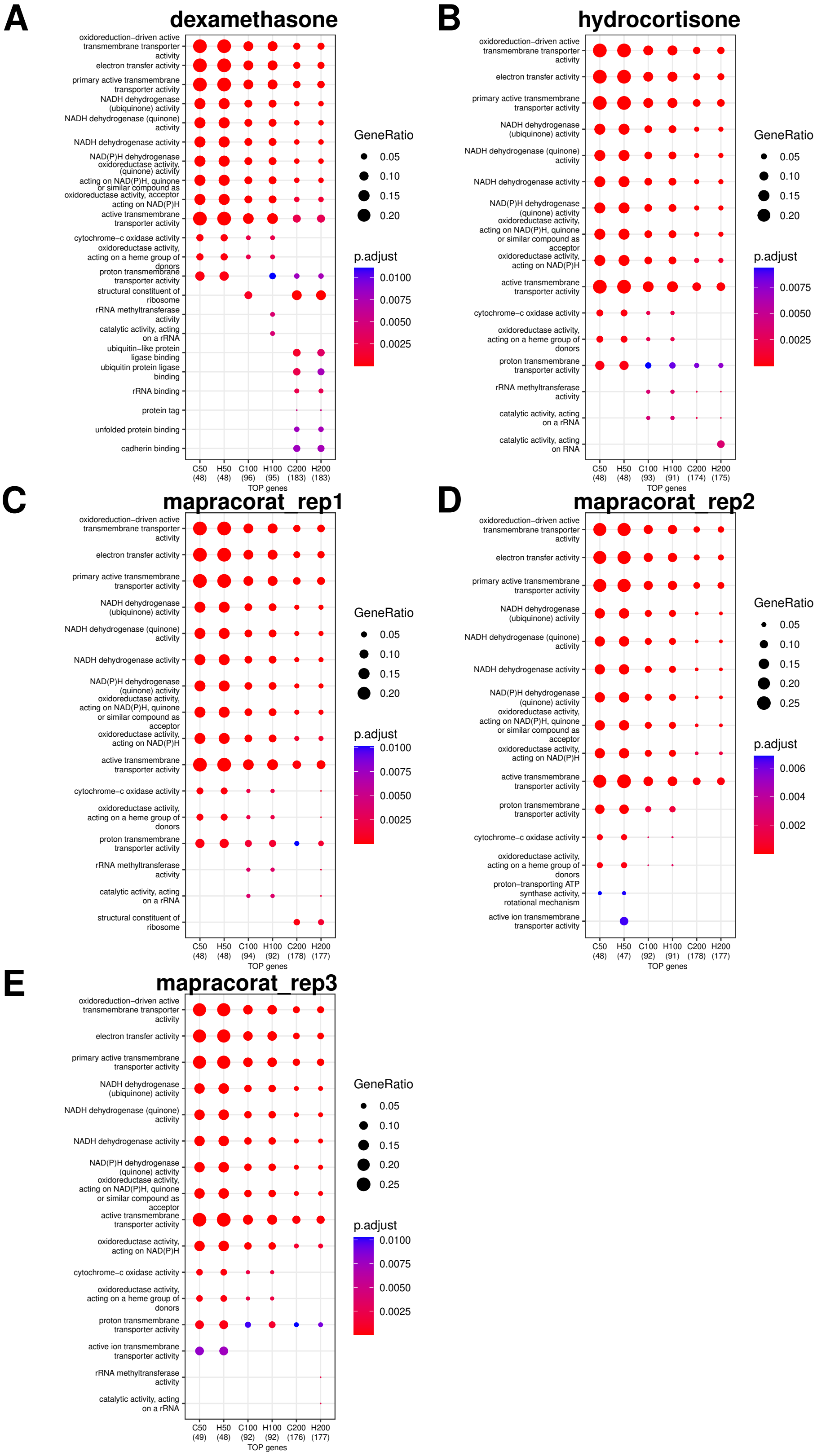

Supplementary Figure 13. Functional underrepresentation by HTSeq-count (--nonunique none) for RNA-seq libraries of dataset 4. Dot plot showing gene ontology (GO) enrichment analysis of the 50, 100, and 200 protein-coding genes with the highest expression values as computed by HTSeq-count using default parameters ("--nonunique none") ("H50", "H100", and "H200", respectively) or our "multimapper-aware" strategy ("C50", "C100", and "C200", respectively). **(A)** Dexamethasone. **(B)** Hydrocortisone. **(C)** Mapracorat – replicate 1. **(D)** Mapracorat – replicate 2. **(E)** Mapracorat – replicate 3. For complete description, see legend of Figure 2C.
